# Sarcopenia Was a Poor Prognostic Predictor for Patients With Advanced Lung Cancer Treated With Immune Checkpoint Inhibitors

**DOI:** 10.3389/fnut.2022.900823

**Published:** 2022-07-18

**Authors:** Shuluan Li, Zhou Liu, Ya Ren, Jinying Liu, Shiqi Lv, Pin He, Yajing Yang, Yanfen Sun, Jianhua Chang, Dehong Luo, Minghua Cong

**Affiliations:** ^1^Department of Nutrition, National Cancer Center, National Clinical Research Center for Cancer, Cancer Hospital and Shenzhen Hospital, Chinese Academy of Medical Sciences, Peking Union Medical College, Shenzhen, China; ^2^Department of Radiology, National Cancer Center, National Clinical Research Center for Cancer, Cancer Hospital and Shenzhen Hospital, Chinese Academy of Medical Sciences, Peking Union Medical College, Shenzhen, China; ^3^Department of Nutrition, National Cancer Center, National Clinical Research Center for Cancer, Cancer Hospital, Chinese Academy of Medical Sciences and Peking Union Medical College, Beijing, China; ^4^Department of Oncology, National Cancer Center, National Clinical Research Center for Cancer, Cancer Hospital and Shenzhen Hospital, Chinese Academy of Medical Sciences, Peking Union Medical College, Shenzhen, China; ^5^Comprehensive Oncology Department, National Cancer Center, National Clinical Research Center for Cancer, Cancer Hospital, Chinese Academy of Medical Sciences, Peking Union Medical College, Beijing, China; ^6^Comprehensive Oncology Department, Hebei Cancer Hospital, Chinese Academy of Medical Sciences, Peking Union Medical College, Beijing, China

**Keywords:** skeletal muscle index (SMI), sarcopenia, advanced lung cancer, immune-checkpoint inhibitor, progression free survival (PFS)

## Abstract

**Background:**

It remains not well known whether skeletal muscle mass (SMM) loss has any impact on the effectiveness of immune checkpoint inhibitors (ICIs) in patients with advanced lung cancer. We aimed to evaluate the association between SMM and clinical outcome of patients with advanced lung cancer receiving ICIs as first line or second line.

**Materials and Methods:**

From March 1st, 2019 to March 31st, 2021 at our hospital, 34 patients with advanced lung cancer treated with first-line or second-line ICIs were enrolled retrospectively. The estimation of skeletal muscle index (SMI) for sarcopenia was assessed at the level of the third lumbar vertebra (L3) on computed tomography (CT) images obtained within 4 weeks before initiation of ICIs treatment. The impact of sarcopenia (low SMI) on progression free survival (PFS) was analyzed using Kaplan-Meier method and log-rank tests. The effect of various variables on PFS was evaluated using Cox proportional hazards regression model with univariate and multivariate analysis. The impact on treatment response including objective response rate (ORR) and disease control rate (DCR) and immunotherapy related adverse events (irAEs) between patients with and without sarcopenia was compared by the chi-squared test. The comparison of SMI value between patients with objective response (OR), disease control (DC) and those without OR and DC was used student *t*-test or Mann-Whitney *U* test.

**Results:**

Both in univariate and multivariate analysis, sarcopenia and treatment lines were the predictive factors for PFS (*p* < 0.05). Patients with sarcopenia had significantly shorter PFS than that of non-sarcopenic ones [6.57 vs. 16.2 months, hazard ratios (HR) = 2.947 and 3.542, and 95% confidence interval (CI): 1.123–13.183 and 1.11–11.308, *p* = 0.022 and 0.033]. No significant difference in ORR and irAEs was found. Patients with sarcopenia had lower DCR than those without sarcopenia. The mean SMI value of DCR group and non-DCR group was 32.94 ± 5.49 and 44.77 ± 9.06 cm^2^/m^2^, respectively (*p* = 0.008).

**Conclusion:**

Sarcopenia before immunotherapy might be a significant predictor for poor prognosis including shorter PFS and lower DCR in patients with advanced lung cancer treated with ICIs as first line or second line.

## Introduction

Lung cancer is the second common malignant tumor and the leading cause of cancer-related deaths worldwide (1). Nearly 70% of patients are at stage IV when initially diagnosed with lung cancer (2), with 5-year survival rate as low as 16% (3). With the introduction of immune checkpoint inhibitors (ICIs), such as PD-1 (programmed cell death protein-1) and PD-L1 (programmed death ligand 1) inhibitors, 2 to 3-year survival rates were increased by 15–20% (4, 5). However, not every eligible candidate could benefit from ICIs. Although PD-L1 expression and tumor mutational burden (TMB) have been reported as potential predictors allowing for therapeutic effect prediction for ICIs, even among lung cancer patients with positive PD-L1 (TPS ≥ 1%) or high expression (TPS ≥ 50%), only 10–20% of patients could benefit from ICIs (6, 7). Therefore, it is essential at present to find more reliable biomarkers that can identify patients who are most likely to benefit from ICIs.

Sarcopenia, characterized by loss of skeletal muscle mass and function (8), has been proposed to be associated with tumor-induced increased protein degradation and decreased protein synthesis caused by impaired Akt–mTORC1 pathway in the presence of disturbed metabolic homeostasis, malnutrition, or reduced activity (9, 10). Sarcopenia has been reported to be associated with treatment efficacy, quality of life and clinical outcomes of lung cancer patients receiving chemotherapy or surgery (11–13). However, is sarcopenia associated with the efficacy of ICIs? To investigate potential association, our team has recently performed a systematic review which showed that the PFS of patients with sarcopenia treated with ICIs was 1.46 times shorter than those without sarcopenia in various types of cancer (14), suggesting that sarcopenia may be a potential predictor for the efficacy of immunotherapy. Besides, skeletal muscle is now recognized as an immune regulatory organ that can regulates immunological processes and the inflammatory response, and the efficacy of ICIs is heavily dependent on the host’s immune system (15). Therefore, sarcopenia is also very likely to be associated with the efficacy of ICIs.

Previously, several studies have attempted to investigate the potential impact of sarcopenia on the efficacy of ICIs, but with inconsistent results (16–30). These results cannot be directly compared, for their different inclusion criteria, different definition of sarcopenia [total cross-skeletal muscle (16–18, 21–25, 27, 29) vs. psoas muscle area (19, 20, 26, 28)], or different methods for measuring muscle mass [CT (16–29) vs. DXA (30)]. In addition, the majority of studies enrolled patients receiving second or more treatment lines ICIs, while only three studies enrolled a small proportion [13.4% (23), 16.5% (17), and 38.3% (30), respectively] of patients receiving ICIs as first-line treatment. Therefore, we designed a retrospective study to investigate the potential impact of sarcopenia on the efficacy of ICIs, in which sarcopenia was defined by averaging muscle area on multiple consecutive CT images at L3 level and a large proportion of patients receiving ICIs as first-line treatment were included.

## Materials and Methods

### Patients

In total, 34 consecutive patients with advanced lung cancer treated with ICIs were enrolled in National Cancer Center/National Clinical Research Center for Cancer/Cancer Hospital and Shenzhen Hospital from March 1, 2019 to March 31, 2021. The inclusion criteria for eligible patients were as follows: (1) Patients were diagnosed with stage IV lung cancer including small cell lung cancer, adenocarcinoma, squamous cell carcinoma, large cell neuroendocrine carcinoma, and other types. (2) Patients were treated with ICIs in our hospital for the first time, including PD-1 or PD-L1 inhibitors or CTLA-4 inhibitors as first-line or second-line. (3) Patients received upper abdomen CT scan within 4 weeks before ICIs therapy in our hospital. And those patients with degraded CT images insufficient to perform measurements of muscle area were also excluded.

### Data Collection and Follow-Up

**FIGURE 1:**
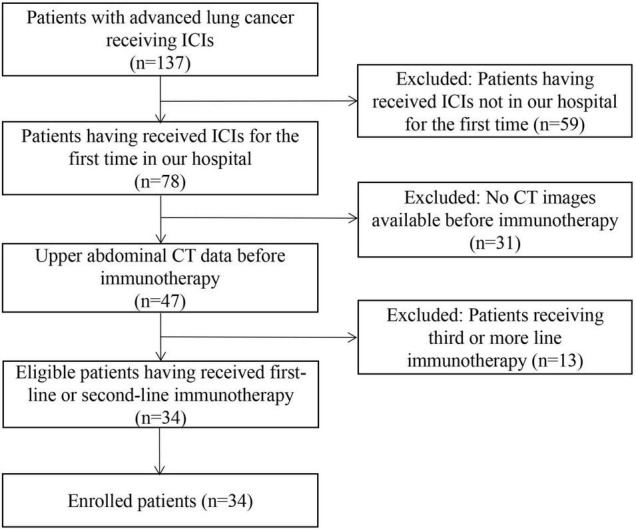
Patient selection flow-chart. ICIs, immune checkpoint inhibitors; CT, computed tomography.

In total, 137 consecutive patients with lung cancer who treated with ICIs from the electronic database of our hospital were identified. After selection based on inclusion and exclusion criteria, 34 eligible patients receiving first-line or second-line immunotherapy were finally included (Figure 1).

The data of all eligible patients were obtained, including age, sex, smoking histology, histopathology, TNM stage, ECOG PS, height and weight at the time of ICI initiation, gene expression status for EGFR and ALK, PD-L1 expression, treatment options, and CT results, treatment response, date of progression defined by CT, and date of the last follow-up. The follow-up date was ended on September 30, 2021.

The primary endpoint was progression free survival (PFS) defined as the time from initiation of immunotherapy to disease progression or death. Secondary endpoints were objective response rate (ORR), disease control rate (DCR) and immunotherapy-related adverse events (irAEs). ORR was defined as the sum of proportion of complete response (CR) and partial response (PR), while DCR was defined as the sum of proportion of CR, PR, and stable disease (SD) according to iRECIST criteria (31). Any reported adverse events that might be associated with immune therapy were obtained from the medical records, mainly including immune pneumonitis, hepatotoxicity, endocrine toxicity, cardiotoxicity, etc.

### Body Composition Analysis

**FIGURE 2:**
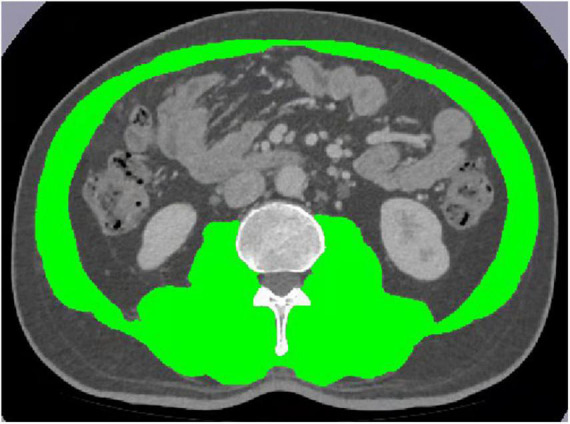
Skeletal muscle mass analysis of computed tomography images on an L3 section by SliceOmatic.

All the enrolled patients underwent CT scan on a GE scanner (Revolution GE, United States) with following parameters: voltage of 140 kvp, tube current of 740 mA and slice thickness of 1.25 mm. The cross-sectional areas of muscle were quantified on CT images acquired within 4 weeks before immunotherapy. All consecutive axial CT slices covering the upper and lower level of the third lumbar vertebra (around 20 slices for each patient) at venous contrast-enhancement phase were chosen. On the platform of sliceOmatic (TomoVision 5.0, Magog, QC, Canada), muscle area (including the psoas, rectus abdominis, transversus abdominis, internal and external abdominal oblique muscles) were firstly manually outlined using morphology mode by YR and PH both with 4 years of experience in abdominal CT imaging and then reviewed by a senior radiologist (ZL with more than 10 years of experience in abdominal CT imaging), who were blinded for patient characteristics (Figure 2). The total volume of skeletal muscle at the level of the third lumbar vertebra was automatically calculated. The average skeletal muscle area of all slices at the level of the third lumbar vertebra was calculated by the following formula: Muscle area = Volume (cm^3^)/(Thickness × Numbers of slice). Skeletal muscle mass (SMM) was defined as the total cross-sectional skeletal muscle area (TMA in cm^2^). SMI was calculated by dividing the total cross-sectional skeletal muscle area (TMA-cm^2^) at the level of lumbar vertebra L3 by height squared (m^2^), which could proportionally reflect whole-body muscles of patients (32, 33). And measurements of muscle on sectional CT is considered as the gold standard method at present (34, 35). Sarcopenia was defined as low SMI as following: (1) for women, SMI < 41 cm^2^/m^2^ regardless of their BMI; (2) for men, SMI < 43 cm^2^/m^2^ with a BMI < 25 kg/m^2^, or SMI < 53 cm^2^/m^2^ with a BMI ≥ 25 kg/m^2^ (36).

### Statistical Analysis

Statistical analysis was performed using SPSS software version 20.0 (IBM, Armonk, NY, United States). The PFS comparison between patients with and without sarcopenia was evaluated using Kaplan-Meier method and log-rank test. The impact of sarcopenia and other factors on PFS were evaluated using univariate and multivariate analysis by Cox proportional hazards regression model. Chi-squared test was used to compare the treatment response and occurrence of irAEs between the sarcopenia and non-sarcopenia groups, while student *t*-test or Mann-Whitney *U* test was used to test any difference in SMI value between patients with objective response, disease control and those without OR and DC. And two-sided *p* < 0.05 was considered to be statistically significant.

## Results

### Patients’ Characteristics

In total, 34 patients were included in the analysis. Baseline characteristics of these patients are shown in Table 1.

**TABLE 1:** Baseline characteristics of the study population.

Characteristics	*N* = 34
**Gender**	
Male, *N* (%)	29 (85.3%)
Female, *N* (%)	5 (14.7%)
**Median age (year), Median ± SD**	63 ± 9.65
**Smoking history**	
Yes, *N* (%)	20 (58.8%)
No, *N* (%)	14 (41.2%)
**BMI (Kg/cm^2^)**	
<18.5, *N* (%)	5 (14.7%)
18.5–24.9, *N* (%)	22 (64.7%)
≥25, *N* (%)	7 (20.6%)
**Histopathology**	
Adenocarcinoma, *N* (%)	23 (67.6%)
Squamous cell carcinoma, *N* (%)	7 (20.6%)
Small cell lung cancer, *N* (%)	3 (8.8%)
Large cell carcinoma, *N* (%)	1 (3.0%)
**Driver gene expression**	
EGFR mutation, *N* (%)	0 (0%)
ALK mutation, *N* (%)	0 (0%)
KRAS mutation, *N* (%)	7 (20.1%)
**PD-L1 expression**	
<1%, *N* (%)	5 (14.7%)
1–49%, *N* (%)	2 (5.9%)
≥50%, *N* (%)	9 (26.5%)
No record available, *N* (%)	18 (52.9%)
**Treatment line**	
First-line immunotherapy, *N* (%)	26 (76.5%)
Second-line immunotherapy, *N* (%)	8 (23.5%)
**SMI (cm^2^/m^2^), mean ± SD**	
Sarcopenic status	44.52 ± 9.56
Sarcopenia, *N* (%)	18 (52.9%)
Non-sarcopenia, *N* (%)	16 (47.1%)

*SD, standard deviation; BMI, body mass index; EGFR, epidermal growth factor receptor; ALK, anaplastic lymphoma kinase; KRAS, Kirsten Rat Sarcoma Viral Oncogene Homolog; PD-L1, programmed death-ligand 1; SMI, skeletal muscle index.*

### The Effect of Sarcopenia on Clinical Outcomes

#### Progression Free Survival

**FIGURE 3:**
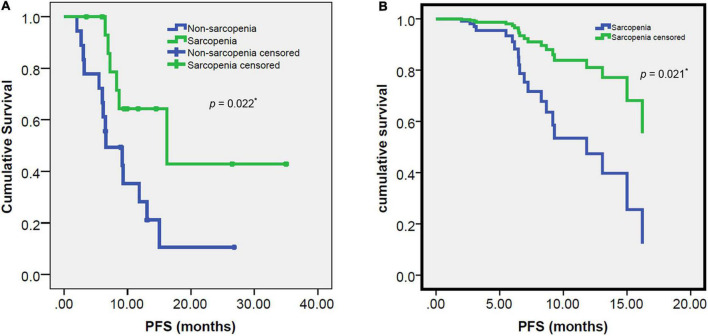
Progression free survival (PFS) survival curves: estimated by Kaplan-Meier method and log-rank test **(A)** and estimated by multivariate analysis of Cox proportional hazards regression model **(B)** for the sarcopenia and non-sarcopenia groups. The PFS was significantly worse in the sarcopenia group than the non-sarcopenia group (6.57 vs. 16.2 months, *p* = 0.022, 0.021, respectively). *Indicates significant difference.

The median follow-up after immunotherapy was 11.67 months (range: 3.50–35.67 months, 95% CI: 8.032–15.308). The median Progression Free Survival (PFS) was 9.3 months (range 2–35 months, 95% CI: 4.817–13.783) for all enrolled 34 patients. The Kaplan-Meier analysis revealed that patients with sarcopenia had a significantly shorter PFS than those without sarcopenia (6.57 vs. 16.2 months, *p* = 0.022; Figure 3A). Among gender, age, smoking history, BMI and PD-L1 expression, sarcopenia and received treatment line, gender, sarcopenia and treatment line were significant prognostic factors for PFS in the univariate analysis (all *p* < 0.05; Table 2). Patients with sarcopenia had a significantly shorter PFS than those without sarcopenia (HR: 2.947, 95% CI: 1.123–7.733, *p* = 0.028).

**TABLE 2:** Univariate and multivariate cox-regression analysis of the risk of sarcopenia and clinicopathological factors on progression free survival (PFS) in patients receiving immune checkpoint inhibitors (ICIs).

Variables		Univariate analysis		Multivariate analysis	
		
		HR (95% CI)	*p*	HR (95% CI)	*p*
Gender	Male	1			
	Female	4.569 (1.599–13.057)	0.005*	2.120 (0.598–7.514)	0.245
Age	–	1.014 (0.970–1.030)	0.546		
Smoking history	No	1			
	Yes	0.488 (0.201–1.185)	0.113		
BMI (kg/cm^2^)	<18.5	1			
	18.5–24.9	0.474 (0.146–1.540)	0.214		
	≥25	0.589 (0.147–2.366)	0.456		
PD-L1 expression	≥50%	1		1	
	<50%	3.073 (0.880–10.730)	0.078	1.947 (0.515–7.365)	0.327
	Unknown	1.24 (0.249–6.268)	0.787	0.608 (0.112–3.295)	0.564
Treatment line	First line	1		1	
	Second line	4.656 (1.698–12.764)	0.003*	9.899 (2.699–36.709)	0.001*
Sarcopenia	Non-low SMI	1		1	
	Low SMI	2.947 (1.123–7.733)	0.028*	4.268 (1.248–14.598)	0.021*

BMI, body mass index; PD-L1, programmed death-ligand 1. *Indicates significant difference.

In multivariate analysis, among gender, PD-L1 expression, sarcopenia and treatment line, treatment line and sarcopenia were independent prognostic factors for PFS (*p* < 0.05; Table 2). The PFS was also significantly shorter in patients with sarcopenia than those without sarcopenia (HR: 4.268, 95% CI: 1.248–14.598, *p* = 0.021; Figure 3B).

#### Objective Response Rate and Disease Control Rate

**FIGURE 4:**
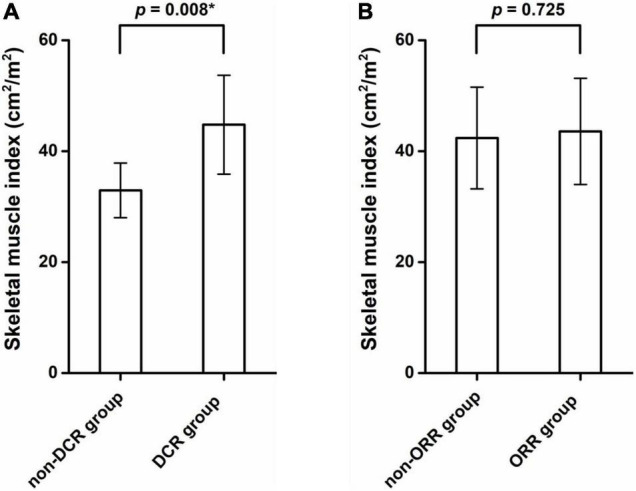
The mean skeletal muscle index (SMI) of the disease control rate (DCR) group was significantly higher than that of the non-DCR group (44.77 ± 9.06 vs. 32.94 ± 5.49 cm^2^/m^2^, *p* = 0.008) **(A)**. No significant difference in SMI values was found between ORR group and non-ORR group (42.37 ± 9.49 vs. 43.56 ± 9.84 cm^2^/m^2^, *p* = 0.725) **(B)**. *Indicates significant difference.

Of 34 patients, 19 patients had objective response with Objective Response Rate (ORR) of 58.8%. There was no significant difference in ORR between sarcopenia and non-sarcopenia groups (44.4 vs. 68.8%, *p* = 0.154). In contrast, Disease Control Rate (DCR) of all patients was 85.3% (29/34). Patients with sarcopenia had a significant lower DCR than those without sarcopenia (72.2 vs. 100%, *p* = 0.022). Specifically, five patients with sarcopenia experienced progressive disease (PD), while none of the patients without sarcopenia experienced PD (Table 3). The mean SMI of the DCR group was significantly higher than that of the non-DCR group, with 44.77 ± 9.06 and 32.94 ± 5.49 cm^2^/m^2^, respectively (*p* = 0.008; Figure 4A). No significant difference was found in SMI between ORR group and non-ORR group (42.37 ± 9.49 vs. 43.56 ± 9.84 cm^2^/m^2^, *p* = 0.725; Figure 4B).

**TABLE 3:** Treatment response including ORR, DCR, and irAEs comparing sarcopenic vs. non-sarcopenic groups.

Variables	CR (*n*)	PR (*n*)	SD (*n*)	PD (*n*)	ORR (%)	DCR (%)	irAEs (any grade)
Sarcopenia	0	8	5	5	44.4	72.2	7
Non-sarcopenia	0	11	5	0	68.8	100	4
χ^2^ value					2.03	5.211	0.747
*P* value					0.154	0.022*	0.388

Chi-Square Test. CR, complete response; PR, partial response; SD, stable disease; PD, progressive disease; ORR, objective response rate; DCR, disease control rate; irAEs, immunotherapy-related adverse events. *Indicates significant difference.

#### Immune-Related Adverse Events

In our study, 11 patients (11/34, 32.4%) experienced Immune-Related Adverse Events (irAEs). Seven cases with irAEs were in sarcopenia group and four cases in non-sarcopenia group, with no statistically significant difference (*p* = 0.388; Table 3). The most frequent irAEs were increased aminotransferase (*n* = 3) and pneumonitis (*n* = 2). Overall, three patients experienced ¾grade irAEs, including arthralgia and myositis (*n* = 1), pneumonitis (*n* = 1) and abnormal aminotransferase (*n* = 1).

## Discussion

This study investigated the prognostic value of sarcopenia in patients with advanced lung cancer treated with first-line or second-line ICIs. We found that patients with sarcopenia receiving ICIs showed significantly shorter PFS than those non-sarcopenic ones. Although ORR and irAEs were not significantly different between patients with and without sarcopenia, sarcopenic patients did show significantly lower DCR than non-sarcopenic patients. And the mean value of SMI was significantly higher in DCR group than that non-DCR group.

In our study, we found that patients with sarcopenia at the initial stage before immunotherapy had significantly shorter PFS than those without sarcopenia. Sarcopenia was shown as a poor prognosis factor of PFS both at univariate and multivariate analysis. Our results were in line with the previous reported result (17, 19, 20, 22, 26, 27, 29, 30), but not with other studies (18, 23, 24, 28). The majority of studies enrolled patients receiving second or more treatment lines ICIs, while only three studies enrolled a small proportion of patients receiving ICIs as first-line treatment (17, 23, 30). By contrast, in our study, 26 (76.5%) patients received ICIs as first-line treatment. The results we obtained were consistent with two studies focused on patients receiving ICIs as first-line treatment (17, 30), but inconsistent with another one (23). We speculate this may be due to different inclusion criteria, different definition of sarcopenia, or different methods for measuring muscle mass. Sarcopenia can be diagnosed by dual-energy X-ray absorptiometry scan, bioelectrical impedance analysis, CT, and magnetic resonance imaging (MRI) (35). In our study, we chose to measure muscle area at the level of L3 using CT, like the majority of studies. However, unlike previous studies using a single or two random slices at the level of L3 (16, 21, 25, 29), we measured 20 consecutive slices at the level of L3 and average the muscle area to decrease the randomness and potential selection bias. Besides, instead of using radiodensity threshold-based segmentation, we used “Morphology mode” of sliceOmatic that segment image not only based on image intensity, but also local morphology of muscle by computing the Watershed of the gradient of the image, which can avoid the bias caused by radiodensity of skeletal muscle.

Also in our study, patients receiving ICIs as first line had significantly longer PFS than those as second line in both univariate and multivariate analysis, suggesting that immunotherapy might be better prescribed as first-line treatment. The similar results have been reported in previous large cohort clinical studies (5, 37). Due to limited cases, we did not perform subgroup analysis on the potential impact of sarcopenia on the first-line treatment group and second-line treatment group, respectively, which warrants further investigation. Partially due to limited number of patients with available PD-L1 testing, our study showed that PD-L1 expression had no positive impact on PFS in the univariate analysis and multivariate analysis, which was inconsistent with previous study (38).

In addition, we found that patients with sarcopenia had significantly lower DCR than those without sarcopenia, and the mean SMI in the DCR group was significantly higher than that in the non-DCR group. The result was in accordance with the previous studies (17, 26, 30), but inconsistent with other study (19, 23, 28). However, for ORR, we found that sarcopenic patients had a trend toward lower ORR, but without significance, which was consistent with some reports (16, 17, 24, 27, 28), but inconsistent with other studies (26). Previous article showed that sarcopenia are associated with the development of hyperprogressive disease after second-line pembrolizumab in patients with non-small-cell lung cancer (39). In terms of potential impact of sarcopenia on DCR and ORR, these inconsistent results in different studies might come from different study design, different inclusion and exclusion criteria, different sample size and different way of measuring muscle area or definition of sarcopenia, etc., which needs further investigation in a prospective study with larger sample size.

In our statistical analysis, sarcopenia was not a significant factor for predicting irAEs, which was similar to our previous report (40) and other studies (16, 18, 27). However, previous study found that patients with sarcopenia experienced significantly increased risk of irAEs (22, 41). It is generally believed that sarcopenia has been associated with a greater incidence of chemotherapy toxicity, but the impact of sarcopenia on irAEs remains controversial. These controversial results from previous reports cannot be directly compared, due to varied number of patients enrolled, inconsistent tools for measuring muscle mass, different definition of sarcopenia, and different treatment regimens including ICIs. A standardized workflow of studying the impact of sarcopenia should be made before sarcopenia as a reliable prognostic biomarker could be translated into clinical practice.

With respect to the underlying mechanism of sarcopenia affecting treatment efficacy of ICIs is still not fully known, multiple studies have proposed chronic inflammation might play a central role in adverse affecting immunotherapy, such as increased neutrophil-to-lymphocyte ratio (NLR), leukocyte/lymphocyte ratio (LLR), red blood cell distribution width (RDW), TGF-α, Fibrinogen and CRP (26, 29, 30). Previous studies found that IL-15 is as a myokine expressed in skeletal muscle cells and regulates CD8 T-cell and promotes survival of T-cells (42, 43), which is important in maintaining body immune function. IL-15 serum levels decrease in older people with loss of muscle mass (44), which suggested that sarcopenia may lead to immune function impaired. And the expression of increased IL-6 and decreased IL-7 in people with loss of muscle mass effects immune system function through T-cell exhaustion (42, 44). CD4 + FoxP3 + Tregs infiltrate impaired skeletal muscle, which suggested that sarcopenia may lead to tumor immune escape (45).

Our study has several limitations. First, it is retrospective study and sample size is relatively small. Second, sarcopenia is merely defined according to SMI, without taking muscle strength and function into account, for example grip. In addition, the cut-off value of SMI was based on literature in white people instead of Asian people. Third, due to short follow-up time, the potential impact of sarcopenia on OS could not be investigated in this study.

In conclusion, sarcopenia may be a poor prognostic factor of patients with advanced lung cancer receiving ICIs as first-line or second-line treatment. Patients with sarcopenia had a significantly lower DCR and shorter PFS than those without sarcopenia, suggesting sarcopenia should be taken into consideration when using ICIs in clinical practice.

## Data Availability Statement

The original contributions presented in this study are included in the article/supplementary material, further inquiries can be directed to the corresponding authors.

## Author Contributions

SLi: idea and original draft preparation, methodology, and review and editing. ZL: methodology and review and editing. YR: visualization and review and editing. JL and YY: supervision. SLv, PH, and YS: data curation. JC: resources. DL: project administration. MC: idea and software. All authors have read and agreed to the published version of the manuscript.

## Conflict of Interest

The authors declare that the research was conducted in the absence of any commercial or financial relationships that could be construed as a potential conflict of interest.

## Publisher’s Note

All claims expressed in this article are solely those of the authors and do not necessarily represent those of their affiliated organizations, or those of the publisher, the editors and the reviewers. Any product that may be evaluated in this article, or claim that may be made by its manufacturer, is not guaranteed or endorsed by the publisher.

## References

[1] Sung H Ferlay J Siegel RL Laversanne M Soerjomataram I Jemal A Global cancer statistics 2020: GLOBOCAN estimates of incidence and mortality worldwide for 36 cancers in 185 Countries. *CA Cancer J Clin.* (2021) 71:209–49. 10.3322/caac.21660 33538338

[2] Knight SB Crosbie PA Balata H Chudziak J Hussell T Dive C. Progress and prospects of early detection in lung cancer. *Open Biol.* (2017) 7:170070. 10.1098/rsob.170070 28878044 PMC5627048

[3] Howlader N Noone AM Krapcho M Miller D Brest A Yu M eds *SEER Cancer Statistics Review, 1975–2016.* Bethesda, MD: National Cancer Institute (2019). 1 p.

[4] Ramalingam SS Ciuleanu TE Pluzanski A Lee J-S Schenker M Bernabe Caro R Nivolumab + ipilimumab versus platinum-doublet chemotherapy as first-line treatment for advanced non-small cell lung cancer: three-year update from CheckMate 227 Part 1. *J Clin Oncol.* (2020) 38:9500. 10.1200/JCO.2020.38.15_suppl.9500

[5] Rodriguez-Abreu D Powell SF Hochmair M Gadgeel SM Esteban E Felip E Final analysis of KEYNOTE-189: pemetrexed-platinum chemotherapy (chemo) with or without pembrolizumab (pembro) in patients (pts) with previously untreated metastatic nonsquamous non-small cell lung cancer (NSCLC). *J Clin Oncol.* (2020) 38:9582. 10.1200/JCO.2020.38.15_suppl.9582 32150489

[6] Reck M Schenker M Lee KH Provencio M Nishio M Lesniewski-Kmak K Nivolumab plus ipilimumab versus chemotherapy as first-line treatment in advanced non–small-cell lung cancer with high tumour mutational burden: patient-reported outcomes results from the randomised, open-label, phase III checkmate 227 trial. *Eur J Cancer.* (2019) 116:137–47. 10.1016/j.ejca.2019.05.008 31195357

[7] Mok TSK Wu Y-L Kudaba I Kowalski DM Cho BC Turna HZ Pembrolizumab versus chemotherapy for previously untreated, PD-L1-expressing, locally advanced or metastatic non-small-cell lung cancer (KEYNOTE-042): a randomised, open-label, controlled, phase 3 trial. *Lancet.* (2019) 393:1819–30. 10.1016/S0140-6736(18)32409-7 30955977

[8] Fielding RA Vellas B Evans WJ Bhasin S Morley JE Newman AB Sarcopenia: an undiagnosed condition in older adults. Current consensus definition: prevalence, etiology, and consequences. International working group on sarcopenia. *J Am Med Dir Assoc.* (2011) 12:249–56. 10.1016/j.jamda.2011.01.003 21527165 PMC3377163

[9] Geremia A Sartori R Baraldo M Nogara L Balmaceda V Dumitras GA Activation of Akt-mTORC1 signalling reverts cancer-dependent muscle wasting. *J Cachexia Sarcopenia Muscle.* (2022) 13:648–61. 10.1002/jcsm.12854 34741441 PMC8818597

[10] Meza-Valderrama D Marco E Dávalos-Yerovi V Muns MD Tejero-Sánchez M Duarte E Sarcopenia, malnutrition, and cachexia: adapting definitions and terminology of nutritional disorders in older people with cancer. *Nutrients.* (2021) 13:761. 10.3390/nu13030761 33652812 PMC7996854

[11] Nipp RD Fuchs G El-Jawahri A Mario J Troschel FM Greer JA Sarcopenia is associated with quality of life and depression in patients with advanced cancer. *Oncologist.* (2018) 23:97–104. 10.1634/theoncologist.2017-0255 28935775 PMC5759817

[12] ChenXHouLShenYWuXDongBHaoQ. The role of baseline sarcopenia index in predicting chemotherapy-induced undesirable effects and mortality in older people with stage III or IV non-small cell lung cancer. *J Nutr Health Aging.* (2021) 25:878–82. 10.1007/s12603-021-1633-3 34409965

[13] Takahashi Y Suzuki S Hamada K Nakada T Oya Y Sakakura N Sarcopenia is poor risk for unfavorable short- and long-term outcomes in stage I non-small cell lung cancer. *Ann Transl Med.* (2021) 9:325. 10.21037/atm-20-4380 33708952 PMC7944314

[14] Li S Wang T Tong G Li X You D Cong M. Prognostic impact of sarcopenia on clinical outcomes in malignancies treated with immune checkpoint inhibitors: a systematic review and meta-analysis. *Front Oncol.* (2021) 11:726257. 10.3389/fonc.2021.726257 34513704 PMC8427761

[15] Afzali AM Müntefering T Wiendl H Meuth SG Ruck T. Skeletal muscle cells actively shape (auto)immune responses. *Autoimmun Rev.* (2018) 17:518–29. 10.1016/j.autrev.2017.12.005 29526638

[16] Cortellini A Verna L Porzio G Bozzetti F Palumbo P Masciocchi C Predictive value of skeletal muscle mass for immunotherapy with nivolumab in non-small cell lung cancer patients: a “hypothesis-generator” preliminary report. *Thorac Cancer.* (2019) 10:347–51. 10.1111/1759-7714.12965 30600905 PMC6360197

[17] Takada K Yoneshima Y Tanaka K Okamoto I Shimokawa M Wakasu S Clinical impact of skeletal muscle area in patients with non-small cell lung cancer treated with anti-PD-1 inhibitors. *J Cancer Res Clin Oncol.* (2020) 146:1217–25. 10.1007/s00432-020-03146-5 32025867 PMC11804506

[18] Haik L Gonthier A Quivy A Gross-Goupil M Veillon R Frison E The impact of sarcopenia on the efficacy and safety of immune checkpoint inhibitors in patients with solid tumours. *Acta Oncol.* (2021) 60:1597–603. 10.1080/0284186X.2021.1978540 34549686

[19] Shiroyama T Nagatomo I Koyama S Hirata H Nishida S Miyake K Impact of sarcopenia in patients with advanced non-small cell lung cancer treated with PD-1 inhibitors: a preliminary retrospective study. *Sci Rep.* (2019) 9:2447. 10.1038/s41598-019-39120-6 30792455 PMC6385253

[20] Tsukagoshi M Yokobori T Yajima T Maeno T Shimizu K Mogi A Skeletal muscle mass predicts the outcome of nivolumab treatment for non-small cell lung cancer. *Medicine.* (2020) 99:e19059. 10.1097/MD.0000000000019059 PMC7035054 32049805

[21] Magri V Gottfried T Di Segni M Urban D Peled M Daher S Correlation of body composition by computerized tomography and metabolic parameters with survival of nivolumab-treated lung cancer patients. *Cancer Manag Res.* (2019) 11:8201–7. 10.2147/CMAR.S210958 31564979 PMC6733251

[22] Strulov Shachar S Fried R Shafran I Moskovitz MT Williams GR Bar-Sela G Body composition as predictor of toxicity and outcomes in patients with metastatic non-small cell lung cancer (mNSCLC) receiving nivolumab (Nivo). *J Clin Oncol.* (2018) 36:e21010.

[23] Roch B Coffy A Jean-Baptiste S Palaysi E Daures J-P Pujol J-L Cachexia - sarcopenia as a determinant of disease control rate and survival in non-small lung cancer patients receiving immune-checkpoint inhibitors. *Lung Cancer.* (2020) 143:19–26. 10.1016/j.lungcan.2020.03.003 32200137

[24] Nishioka N Naito T Notsu A Mori K Kodama H Miyawaki E Unfavorable impact of decreased muscle quality on the efficacy of immunotherapy for advanced non-small cell lung cancer. *Cancer Med.* (2021) 10:247–56. 10.1002/cam4.3631 33300678 PMC7826480

[25] Degens JHRJ Dingemans AMC Willemsen ACH Gietema HA Hurkmans DP Aerts JG The prognostic value of weight and body composition changes in patients with non-small-cell lung cancer treated with nivolumab. *J Cachexia Sarcopenia Muscle.* (2021) 12:657–64. 10.1002/jcsm.12698 33951326 PMC8200425

[26] Nishioka N Uchino J Hirai S Katayama Y Yoshimura A Okura N Association of sarcopenia with and efficacy of anti-PD-1/PD-L1 therapy in non-small-cell lung cancer. *J Clin Med.* (2019) 8:450. 10.3390/jcm8040450 30987236 PMC6518257

[27] Cortellini A Bozzetti F Palumbo P Brocco D Di Marino P Tinari N Weighing the role of skeletal muscle mass and muscle density in cancer patients receiving PD-1/PD-L1 checkpoint inhibitors: a multicenter real-life study. *Sci Rep.* (2020) 10:1456. 10.1038/s41598-020-58498-2 31996766 PMC6989679

[28] Minami S Ihara S Tanaka T Komuta K. Sarcopenia and visceral adiposity did not affect efficacy of immune-checkpoint inhibitor monotherapy for pretreated patients with advanced non-small cell lung cancer. *World J Oncol.* (2020) 11:9–22. 10.14740/wjon1225 32095185 PMC7011908

[29] Wang Y Chen P Huang J Liu M Peng D Li Z Assessment of sarcopenia as a predictor of poor overall survival for advanced non-small-cell lung cancer patients receiving salvage anti-PD-1 immunotherapy. *Ann Transl Med.* (2021) 9:1801. 10.21037/atm-21-6578 35071495 PMC8756219

[30] Tenuta M Gelibter A Pandozzi C Sirgiovanni G Campolo F Venneri MA Impact of sarcopenia and inflammation on patients with advanced non-small cell lung cancer (NCSCL) treated with immune checkpoint inhibitors (ICIs): a prospective study. *Cancers (Basel).* (2021) 13:6355. 10.3390/cancers13246355 34944975 PMC8699333

[31] Seymour L Bogaerts J Perrone A Ford R Schwartz LH Mandrekar S iRECIST: guidelines for response criteria for use in trials testing immunotherapeutics. *Lancet Oncol.* (2017) 18:e143–52. 10.1016/S1470-2045(17)30074-8 28271869 PMC5648544

[32] Fearon K Strasser F Anker SD Bosaeus I Bruera E Fainsinger RL Definition and classification of cancer cachexia: an international consensus. *Lancet Oncol.* (2011) 12:489–95. 10.1016/S1470-2045(10)70218-7 21296615

[33] Vangelov B Bauer J Moses D Smee R. The effectiveness of skeletal muscle evaluation at the third cervical vertebral level for computed tomography-defined sarcopenia assessment in patients with head and neck cancer. *Head Neck.* (2022) 44:1047–56. 10.1002/hed.27000 35138008 PMC9305498

[34] Martin L Tom M Basualdo-Hammond C Baracos V Gramlich L. Piloting a training program in computed tomography (CT) skeletal muscle assessment for Registered Dietitians. *JPEN J Parenter Enter Nutr.* (2022). 10.1002/jpen.2348 35147237

[35] Cruz-Jentoft AJ Baeyens JP Bauer JM Boirie Y Cederholm T Landi F Sarcopenia: European consensus on definition and diagnosis: report of the European working group on sarcopenia in older people. *Age Ageing.* (2010) 39:412–23. 10.1093/ageing/afq034 20392703 PMC2886201

[36] Martin L Birdsell L Macdonald N Reiman T Clandinin MT McCargar LJ Cancer cachexia in the age of obesity: skeletal muscle depletion is a powerful prognostic factor, independent of body mass index. *J Clin Oncol.* (2013) 31:1539–47. 10.1200/JCO.2012.45.2722 23530101

[37] West H McCleod M Hussein M Morabito A Rittmeyer A Conter HJ Atezolizumab in combination with carboplatin plus nab-paclitaxel chemotherapy compared with chemotherapy alone as first-line treatment for metastatic non-squamous non-small-cell lung cancer (IMpower130): a multicentre, randomised, open-label, phase 3 tria. *Lancet Oncol.* (2019) 20:924–37. 10.1016/S1470-2045(19)30167-6 31122901

[38] Nosaki K Saka H Hosomi Y Baas P de Castro G Jr. Reck M Safety and efficacy of pembrolizumab monotherapy in elderly patients with PD-L1–positive advanced non–small-cell lung cancer: pooled analysis from the KEYNOTE-010, KEYNOTE-024, and KEYNOTE-042 studies. *Lung Cancer.* (2019) 135:188–95. 10.1016/j.lungcan.2019.07.004 31446994

[39] Petrova MP Donev IS Radanova MA Eneva MI Dimitrova EG Valchev GN Sarcopenia and high NLR are associated with the development of hyperprogressive disease after second-line pembrolizumab in patients with non-small-cell lung cancer. *Clin Exp Immunol.* (2020) 202:353–62. 10.1111/cei.13505 32757277 PMC7670147

[40] Li S Wang T Lai W Zhang M Cheng B Wang S Prognostic impact of sarcopenia on immune-related adverse events in malignancies received immune checkpoint inhibitors: a systematic review and meta-analysis. *Transl Cancer Res.* (2021) 10:5150–8. 10.21037/tcr-21-1470 35116365 PMC8797877

[41] Hirsch L Bellesoeur A Boudou-Rouquette P Arrondeau J Thomas-Schoemann A Kirchgesner J The impact of body composition parameters on severe toxicity of nivolumab. *Eur J Cancer.* (2020) 124:170–7. 10.1016/j.ejca.2019.11.003 31794927

[42] Crane JD MacNeil LG Lally JS Ford RJ Bujak AL Brar IK Exercise-stimulated interleukin-15 is controlled by AMPK and regulates skin metabolism and aging. *Aging Cell.* (2015) 14:625–34. 10.1111/acel.12341 25902870 PMC4531076

[43] Conlon KC Lugli E Welles HC Rosenberg SA Fojo AT Morris JC Redistribution, hyperproliferation, activation of natural killer cells and CD8 T cells, and cytokine production during first-in-human clinical trial of recombinant human interleukin-15 in patients with cancer. *J Clin Oncol.* (2015) 33:74–82. 10.1200/JCO.2014.57.3329 25403209 PMC4268254

[44] Duggal NA Pollock RD Lazarus NR Harridge S Lord JM. Major features of immunesenescence, including reduced thymic output, are ameliorated by high levels of physical activity in adulthood. *Aging Cell.* (2018) 17:e12750. 10.1111/acel.12750 29517845 PMC5847865

[45] Saini J McPhee JS Al-Dabbagh S Stewart CE Al-Shanti N. Regenerative function of immune system: modulation of muscle stem cells. *Ageing Res Rev.* (2016) 27:67–76. 10.1016/j.arr.2016.03.006 27039885

